# Nuclear fragments of the neural cell adhesion molecule NCAM with or without polysialic acid differentially regulate gene expression

**DOI:** 10.1038/s41598-017-14056-x

**Published:** 2017-10-19

**Authors:** Nina Westphal, Thomas Theis, Gabriele Loers, Melitta Schachner, Ralf Kleene

**Affiliations:** 1 Zentrum für Molekulare Neurobiologie, Universitätsklinikum Hamburg-Eppendorf, Falkenried 94, 20251 Hamburg, Germany; 2 Center for Neuroscience, Shantou University Medical College, 22 Xin Ling Road, Shantou, Guangdong, 515041 China; 3 Keck Center for Collaborative Neuroscience and Department of Cell Biology and Neuroscience, Rutgers University, 604 Allison Road, Piscataway, NJ 08854 USA

## Abstract

The neural cell adhesion molecule (NCAM) is the major carrier of polysialic acid (PSA) which modulates NCAM functions of neural cells at the cell surface. In previous studies, we have shown that stimulation of cultured neurons with surrogate NCAM ligands leads to the generation and nuclear import of PSA-lacking and -carrying NCAM fragments. Here, we show that the nuclear import of the PSA-carrying NCAM fragment is mediated by positive cofactor 4 and cofilin, which we identified as novel PSA-binding proteins. In the nucleus, the PSA-carrying NCAM fragment interacts via PSA with PC4 and cofilin, which are involved in RNA polymerase II-dependent transcription. Microarray analysis revealed that the nuclear PSA-carrying and -lacking NCAM fragments affect expression of different genes. By qPCR and immunoblot analysis we verified that the nuclear PSA-carrying NCAM fragment increases mRNA and protein expression of nuclear receptor subfamily 2 group F member 6, whereas the PSA-lacking NCAM fragment increases mRNA and protein expression of low density lipoprotein receptor-related protein 2 and α-synuclein. Differential gene expression evoked by nuclear NCAM fragments without and with PSA indicates that PSA-carrying and -lacking NCAM play different functional roles in the nervous system.

## Introduction

NCAM plays important roles in neural cell migration, neurite outgrowth and fasciculation, synaptogenesis and synaptic plasticity, and it is associated with certain forms of emotional behaviour^1–4^. In mammals, NCAM is the predominant carrier of PSA, a polymer of α2,8-linked sialic acid monomers. NCAM and its associated glycan PSA regulate cell interactions during development, affect synaptic activities and regeneration after injury in the adult nervous system, and are implicated in neurodegenerative and psychiatric disorders^5–17^. PSA differentially modulates the functions of the protein backbone of NCAM and plays an important role in regulating the circadian rhythm^18–24^.

In previous studies, we have found that PSA-lacking and -carrying proteolytic NCAM fragments comprising the intracellular and transmembrane domains as well as part of the extracellular domain enter the cell nucleus after their generation at the plasma membrane^25,26^. Calmodulin mediates the nuclear import of the PSA-lacking fragment and associates with this NCAM fragment in the nucleus^25^. The nuclear PSA-carrying NCAM fragment alters clock-related gene expression *in vitro* and the nuclear PSA levels correlate negatively with clock-related gene expression *in vivo* ^26^. In the nucleus, the PSA-carrying NCAM fragment interacts with histone H1 via PSA and both the PSA-carrying and -lacking NCAM fragments interact with histone H1 via their intracellular protein domain^26^.

In the present study, we searched for nuclear PSA-binding proteins and identified positive cofactor 4 (PC4) (also known as activated RNA polymerase II transcriptional coactivator p15, SUB1 homolog and p14) and cofilin (non-muscle specific isoform of cofilin; cofilin-1) as PSA-binding proteins. We provide evidence that PC4 and cofilin mediate the import of the PSA-carrying NCAM fragment and interact with the PSA-carrying NCAM fragment in nuclei of cultured cerebellar neurons. Cofilin is well characterized as actin-binding protein in the cytoplasm^27,28^ and also enters the nucleus and mediates the transport of actin into the nucleus^29^, where it affects the elongation phase of RNA polymerase II-dependent transcription^30^. PC4^31,32^, a non-histone component of chromatin, plays important roles in chromatin organization, DNA replication, DNA repair, and activation of RNA polymerase II-dependent transcription^31–37^.

Here, we show that the nuclear PSA-carrying NCAM fragment up-regulates mRNA and protein expression of nuclear receptor subfamily 2 group F member 6 (Nr2f6) and that the PSA-lacking NCAM fragment up-regulates mRNA and protein expression of low density lipoprotein receptor-related protein 2 (Lrp2) (also known as megalin and gp330) and α-synuclein (Snca). The distinct gene expression by the nuclear PSA-carrying and PSA-lacking NCAM fragments suggests that the function of the NCAM fragment with PSA differs from that of the NCAM fragment without PSA.

## Results

### Cofilin and PC4 are novel PSA binding partners which contribute to the nuclear import of PSA-NCAM

To search for nuclear PSA-binding proteins, we used an alkaline-solubilized nuclear protein fraction from adult mouse brains for immunoaffinity chromatography with PSA-mimicking anti-idiotypic ScFv antibody reacting with the antigen-binding site of monoclonal PSA-specific antibody 735 and thus recognizing a sequence in the antibody 735 that contains motifs for potential binding partners/receptors of PSA^38,39^. After SDS-PAGE and silver staining of the gel, several protein bands were observed in the eluate (Supplementary Fig. S1). Bands of ~32, ~26, ~23, ~18, and ~14 kDa which were not or hardly detectable in the scFv antibody preparation (Supplementary Fig. S1) were subjected to electrospray ionization with tandem mass spectrometry (MS/MS). The mass spectrometric analysis of the ~14 and ~18 kDa protein bands revealed MS/MS spectra of 1258.0 and 1336.6 Da precursor masses (detected as doubly charged ion at m/z = 630.8 and 669.3) that matched the tryptic peptides EQISDIDDAVR (1260.6 Da) of mouse PC4 and YALYDATYETK (1337.6 Da) of mouse cofilin. Masses of the ~32 kDa protein band matched tryptic peptides of histone H1 which was already identified as PSA-binding protein^38^, while masses of the ~23 and ~26 kDa protein bands could not be assigned to a certain protein.

**Figure 1:**
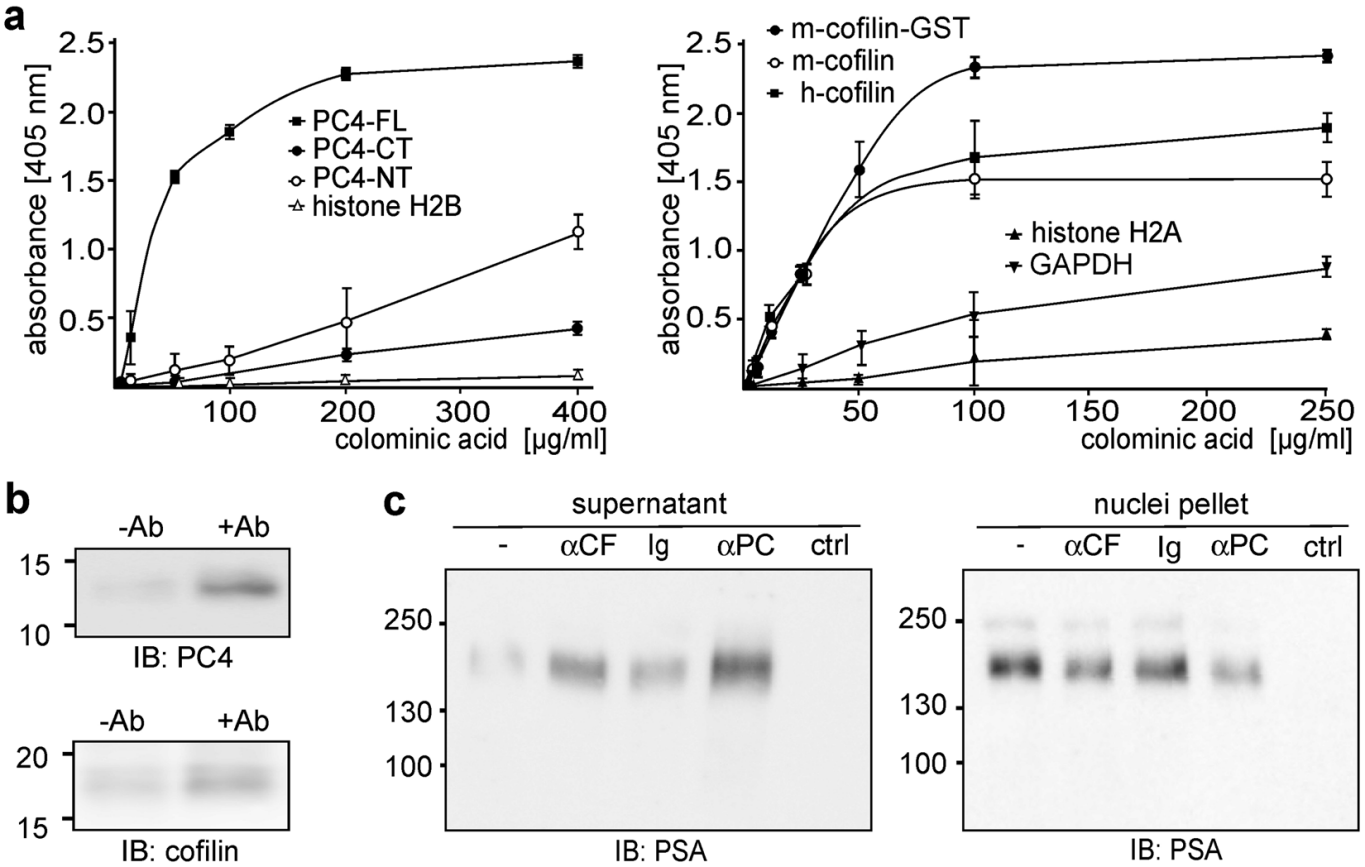
Cofilin and PC4 bind to PSA and mediate the nuclear import of the PSA-carrying NCAM fragment. (**a**) Human (h-cofilin) and mouse (m-cofilin) untagged and mouse GST-tagged (m-cofilin-GST) cofilin as well as full-length (PC4-FL) and N- or C-terminally (PC4-NT, PC4-CT) truncated PC4 were substrate-coated and incubated with increasing concentrations of colominic acid. As negative controls histone H2A and 2B as well as glyceraldehyde 3-phosphate dehydrogenase were used for coating. The binding of colominic acid was detected by ELISA using PSA antibody and HRP-conjugated secondary antibody. Mean values and standard deviations from one representative experiment out of three independent experiments carried out in triplicates are shown. (**b**) Nuclear fractions from wild-type cerebellar neurons treated without (−Ab) or with chicken NCAM antibody (+Ab) were subjected to immunoprecipitation with PSA antibody followed by immunoblot analysis of the immunoprecipitates with cofilin-1 or PC4 antibodies. (**c**) Nuclei were isolated from NCAM-deficient cerebellar neurons and a cytoplasmic fraction was isolated from wild-type cerebellar neurons after treatment with NCAM antibody. Nuclei were incubated with buffer (ctrl) or with the cytoplasmic fraction in the absence (−) or presence of a cofilin (αCF), PC4 (αPC) or control (Ig) antibody followed by centrifugation at 1,000 × g. The pellets and supernatants were subjected to immunoblot (IB) analysis with PSA antibody. (**b, c**) Representative immunoblots out of two independent experiments are shown. Only the ~14 kDa PC4 band and the ~18 kDa cofilin band (**b**) and the upper parts of the blots with PSA-positive bands (**c**) are shown.

By ELISA we showed that colominic acid, which is the bacterial homolog of PSA, bound to substrate-coated mouse and human cofilin as well as to full-length PC4 in a concentration-dependent and saturable manner (Fig. 1a). In contrast, colominic acid did not bind to the nuclear protein histone H2A and it showed only unspecific binding to histone H2B, to the cytoplasmic protein glyceraldehyde 3-phosphate dehydrogenase and to N- or C-terminally truncated PC4 (Fig. 1a). These results confirm that cofilin and PC4 are PSA-binding proteins.

In agreement with these results, PC4 and cofilin were detectable in PSA immunoprecipitates from nuclear fractions of cultured cerebellar neurons after NCAM antibody stimulation, while only low PC4 and cofilin amounts were found in PSA immunoprecipitates from nuclear fractions of non-stimulated neurons (Fig. 1b), showing that the PSA-carrying fragment interacts with cofilin and PC4 in the nucleus.

**Figure 2:**
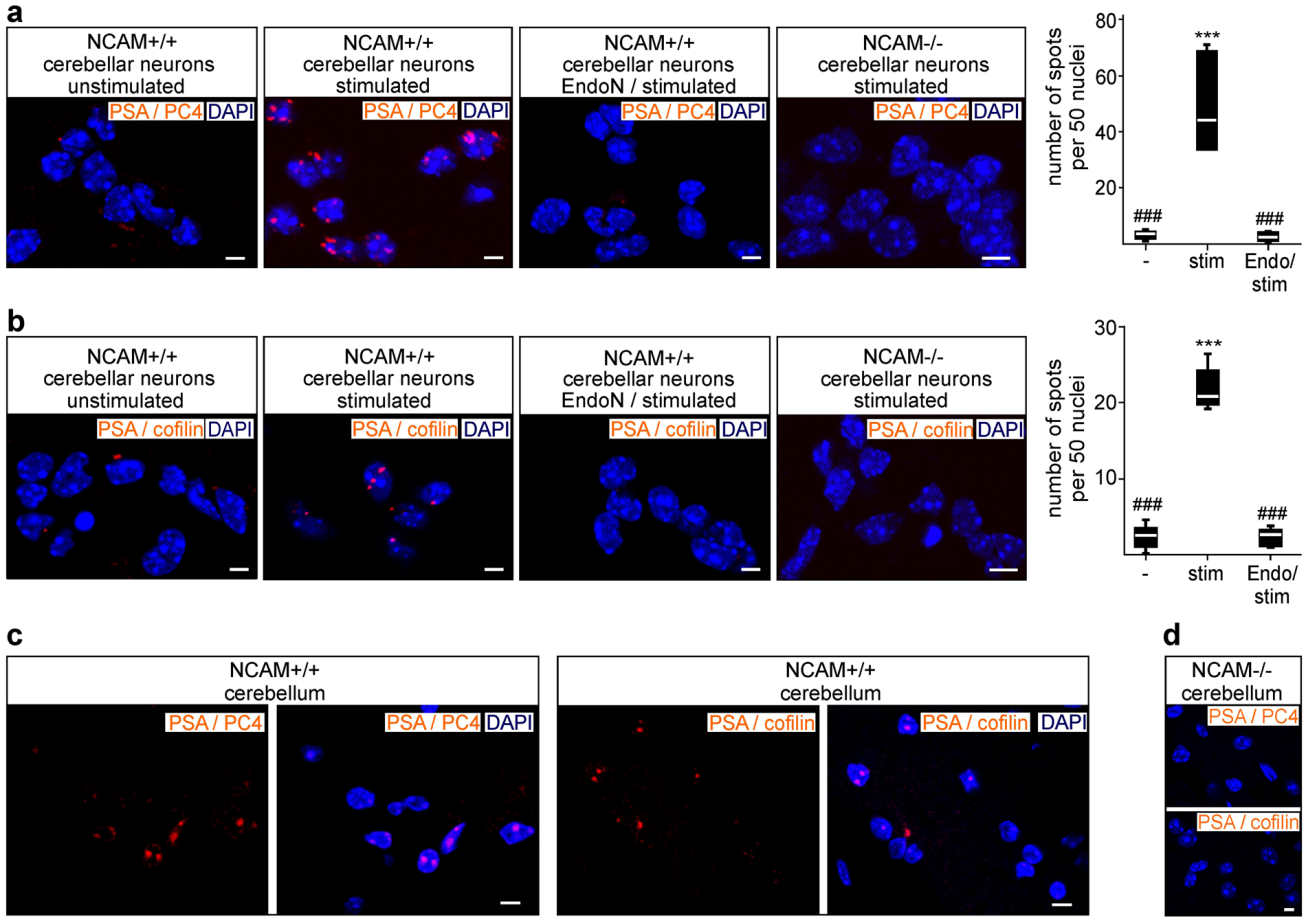
Cofilin and PC4 interact with PSA in nuclei of cerebellar neurons in culture and in tissue sections. (**a**, **b**) Cerebellar neurons from wild-type (NCAM+/+) or NCAM-deficient (NCAM−/−) mice were not treated (unstimulated) or treated with chicken NCAM antibody after pretreatment without (stimulated) or with EndoN (EndoN / stimulated) and subjected to proximity ligation assay with PSA antibody and PC4 antibody (**a**) or cofilin-1 antibody (**b**). Representative confocal fluorescent images of neuronal nuclei are shown. Boxplots are shown for the quantification of nuclear PSA-positive spots by counting the numbers of PSA/PC4-positive (**a**) or PSA/cofilin-positive (**b**) red spots in 50 nuclei of two images per condition from three independent experiments. Differences for stimulated (stim) relative to unstimulated (−) (***p < 0.001) and for EndoN-pretreated stimulated (Endo/stim) and unstimulated (−) neurons relative to stimulated (###p < 0.001) neurons are indicated (two-way ANOVA with Bonferroni post-hoc test). (**c**, **d**) Cerebella from 3-month-old wild-type (NCAM+/+) (**c**) and NCAM-deficient (NCAM−/−) (**d**) mice were subjected to proximity ligation assay with antibodies against PSA and PC4 or cofilin. Representative confocal images of neuronal nuclei are shown. (**a**–**d**) Nuclei were stained with DAPI (blue). Spots of intense fluorescent signals (red) indicate close protein interactions between PSA and PC4 or cofilin. Scale bars: 10 µm.

To analyse whether PSA-carrying NCAM interacts with cofilin or PC4 in neuronal nuclei, proximity ligation assays allowing a highly sensitive and specific detection of close interactions (<40 nm) were performed on cultured cerebellar neurons and tissue slices using antibodies against PSA and cofilin or PC4. Many PSA/PC4- and PSA/cofilin-positive fluorescent signals were observed as spots in the nuclei of cultured cerebellar neurons after stimulation with NCAM antibody, whereas no spots were observed in nuclei of non-stimulated neurons or in NCAM-stimulated neurons after removal of PSA by pretreatment with PSA-degrading endoneuraminidase N (Endo N) (Fig. 2a,b). PSA/PC4- and PSA/cofilin-positive fluorescent spots were also detectable in cerebellar tissue slices from wild-type mice (Fig. 2c), whereas no spots were observed in slices from NCAM-deficient mice (Fig. 2d). These results indicate that PC4 and cofilin associate with the PSA-carrying NCAM fragment in neuronal nuclei.

To test whether cofilin or PC4 are involved in the nuclear import of the PSA-carrying NCAM fragment, a cytoplasmic fraction from NCAM antibody-stimulated wild-type neurons was incubated with nuclei from NCAM-deficient neurons in the absence or presence of cofilin, PC4 or non-immune control antibodies. After incubation and centrifugation for separation of nuclei (pellet) and cytoplasmic fractions (supernatant), the PSA-NCAM levels in the supernatant and pellet fractions in the presence of cofilin or PC4 antibodies were higher and lower, respectively, when compared to the levels in the absence of cofilin or PC4 antibodies and presence of non-immune control antibody (Fig. 1c). This result indicates that cofilin and PC4 antibodies block the nuclear import of PSA-NCAM from the cytoplasm into the nucleus and it suggests that cofilin and PC4 contribute to the import of the PSA-carrying NCAM fragment.

### The nuclear PSA-carrying and PSA-lacking NCAM fragment are involved in expression of different genes

**Figure 3:**
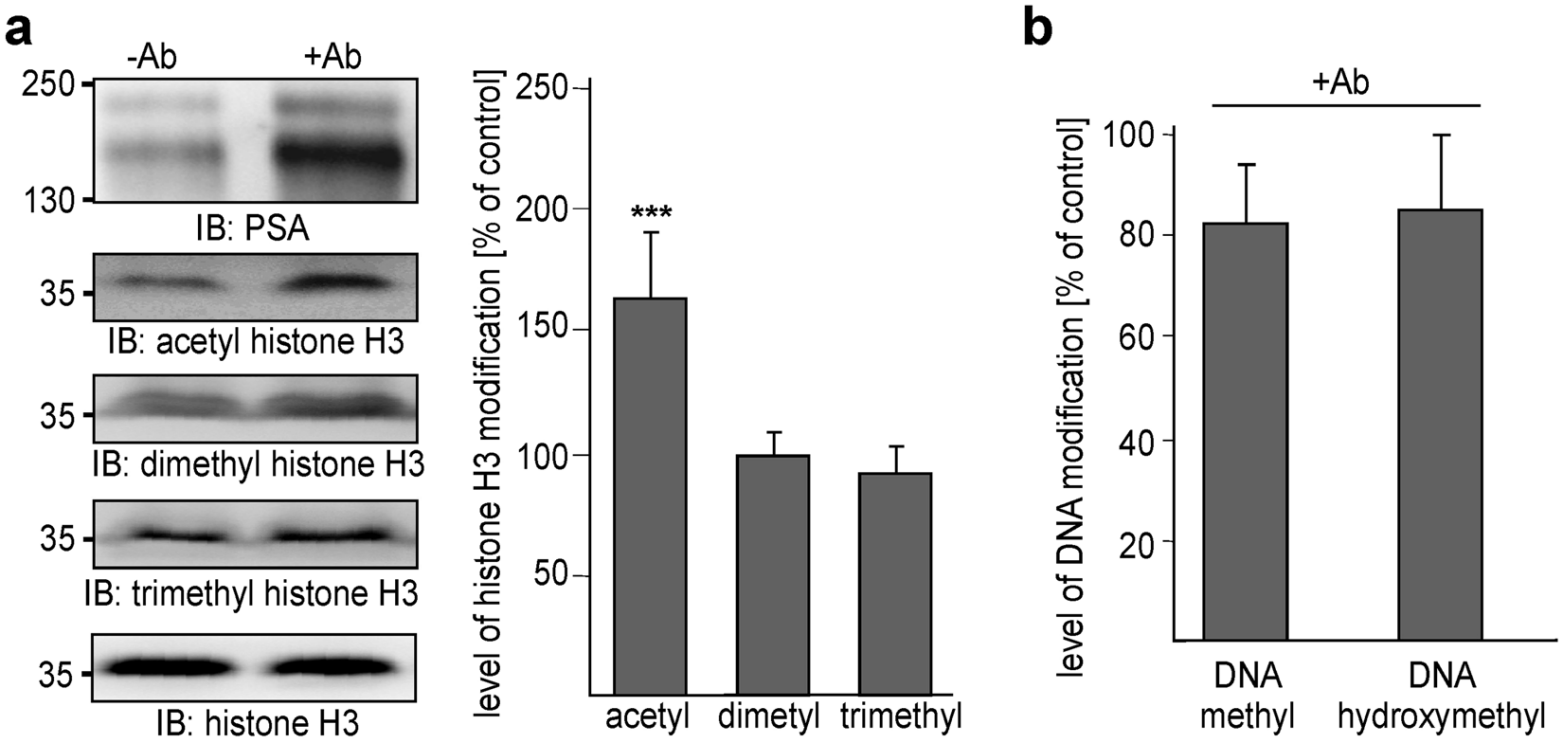
Acetylation of histone H3 is enhanced by treatment of cerebellar neurons with NCAM antibody. Cerebellar neurons were incubated without (−Ab) or with chicken NCAM antibody (+Ab). (**a)** Nuclear fractions were subjected to immunoblot (IB) analysis with antibodies against PSA or histone H3 or with antibodies against acetylated, dimethylated or trimethylated histone H3. Representative immunoblots out of 6 experiments are shown. For clarity only the PSA-positive band and the histone H3-positive bands are shown. Levels of acetylated, dimethylated or trimethylated histone H3 were quantified by densitometry and normalized to total histone H3 levels. Mean values and standard deviations from 6 independent experiments are shown for the levels of acetylated (acetyl), dimethylated (dimethyl) and trimethylated (trimethyl) histone H3 in stimulated neurons relative to the levels in non-treated cells (set to 100%). Differences between groups are indicated (***p < 0.001; two-way ANOVA with Bonferroni post hoc test). (**b)** Cell lysates were subjected to determination of DNA methylation and hydroxymethylation. Mean values and standard deviations from 6 independent experiments are shown for the levels of methylated DNA (DNA methyl) and hydroxymethylated DNA (DNA hydroxymethyl) in stimulated cells relative to the level in non-treated cells (set to 100%).

To gain insights into the nuclear functions of the PSA-lacking and -carrying NCAM fragments in cerebellar neurons, we first analysed whether DNA methylation, histone H3 acetylation and/or histone H3 methylation, which are involved in regulation of transcriptional activation or gene silencing^40–42^, were affected by stimulation with NCAM antibody. In comparison to unstimulated neurons, acetylation of histone H3 at lysine 9 and 14 was increased in neurons after NCAM antibody treatment, whereas trimethylation or dimethylation of histone H3 at lysine 9 as well as levels of methylated and hydroxymethylated DNA were not altered by the NCAM antibody treatment (Fig. 3a,b).

Since enhanced histone H3 acetylation is associated with increased gene expression^40–42^, it is likely that the nuclear PSA-carrying and/or -lacking NCAM fragments are involved in regulating gene expression. The notion that the PSA-carrying NCAM fragment may participate in regulation of gene expression is supported by the finding that nuclear PSA-NCAM interacts with PC4 and cofilin which both have been reported to play a role in RNA polymerase II-dependent transcription^30,37^.

Hypothesizing that differential gene expression is a plausible function of PSA-lacking and PSA-carrying NCAM fragments in the nucleus, we searched for genes which are regulated in their expression by nuclear PSA-lacking and/or PSA-carrying fragments and performed expression profiling using mRNA from cultures of dissociated cerebellar neurons after pretreatment without or with EndoN and treatment without or with NCAM antibody. If genes depend in their expression on the PSA-carrying NCAM fragment, their expression levels should be altered in neurons after NCAM antibody treatment to a similar extent in comparison to the levels in non-treated neurons and to the levels in NCAM antibody-treated neurons after EndoN pre-treatment that specifically removes PSA. However, if genes depend in their expression on the PSA-lacking NCAM fragment, the expression levels of these genes should be altered to a similar extent in neurons after NCAM antibody treatment and in neurons treated with NCAM antibody after pre-treatment with EndoN in comparison to the levels in non-treated neurons. In this view, we found that several genes appeared to be up- or down-regulated in their expression either by the PSA-carrying NCAM fragment (Supplementary Table S1 and Table S2) or by the PSA-lacking NCAM fragment (Supplementary Table S3 and Table S4). These results suggest that the nuclear PSA-lacking and -carrying NCAM fragments are involved in the expression of different genes.

In an independent set of expression profiling experiments with the aim to identify NCAM-specific gene expression triggered by NCAM antibody treatment relative to gene expression induced by control antibodies or antibodies against other cell adhesion molecules, such as L1 and its close homolog CHL1, cultured cerebellar neurons were treated with non-immune control antibodies and with NCAM, CHL1 or L1 antibodies in the absence or presence of the serine protease inhibitor aprotinin, which blocks the generation of the nuclear PSA-lacking NCAM fragment^25^ and of a nuclear L1 fragment^43^. Interestingly, the expression of Nr2f6, Lrp2 and Snca was increased by treatment of cultured cerebellar neurons with the NCAM antibody relative to the treatments with L1, CHL1 and non-immune control antibodies (Table 1), while we did not detect such a NCAM-specific regulation of expression for any of the other genes listed in Supplementary Tables S1, S2, S3 or S4. Of note, Lrp2 and Snca levels were not enhanced by NCAM antibody treatment in the presence of aprotinin (Table 1), supporting the notion that Lrp2 and Snca expression is up-regulated by the nuclear PSA-lacking NCAM fragment.

**Table 1:** Differential gene expression by PSA-lacking and -carrying NCAM fragments. The table shows the relative increase of Nr2f6, Lrp2 and Snca mRNA levels in cerebellar neurons after different treatments performed in microarray 1 or 2 (+apro, aprotinin; chNCAM-Ab, chicken NCAM antibody; rbNCAM-Ab, rabbit NCAM antibody; rbL1-Ab, rabbit L1 antibody; rL1-Ab, rat L1 antibody; rbCHL1-Ab, rabbit CHL1 antibody; rbIg, non-immune rabbit control antibody; rIg, non-immune rat control antibody; EndoN/chNCAM-Ab, chicken NCAM antibody after EndoN pretreatment).

microarray	treatment	Nr2f6	Lrp2	Snca
1	chNCAM-Ab vs untreated	1.320 ± 0.023	1.415 ± 0.021	1.251 ± 0.010
1	chNCAM-Ab vs EndoN/chNCAM-Ab	1.349 ± 0.025	—	—
1	EndoN/chNCAM-Ab vs untreated	—	1.267 ± 0.011	1.249 ± 0.021
2	rbNCAM-Ab vs rbCHL1-Ab	1.425 ± 0.183	1.965 ± 0.247	1.275 ± 0.025
2	rbNCAM-Ab vs rbL1-Ab	1.595 ± 0.098	1.524 ± 0.215	1.505 ± 0.132
2	rbNCAM-Ab vs rIg	2.205 ± 0.256	1.313 ± 0.379	1.284 ± 0.022
2	rbNCAM-Ab vs rL1-Ab	1.915 ± 0.154	1.639 ± 0.060	1.228 ± 0.098
2	rbNCAM-Ab vs rL1-Ab + apro	2.112 ± 0.366	1.496 ± 0.179	1.248 ± 0.071
2	rbNCAM-Ab vs rbNCAM-Ab + apro	0.908 ± 0.079	1.800 ± 0.039	1.246 ± 0.070

### The nuclear PSA-carrying NCAM fragment regulates mRNA and protein expression of Nr2f6, whereas the nuclear PSA-lacking NCAM fragment regulates mRNA and protein expression of Lrp2 and Snca

**Figure 4:**
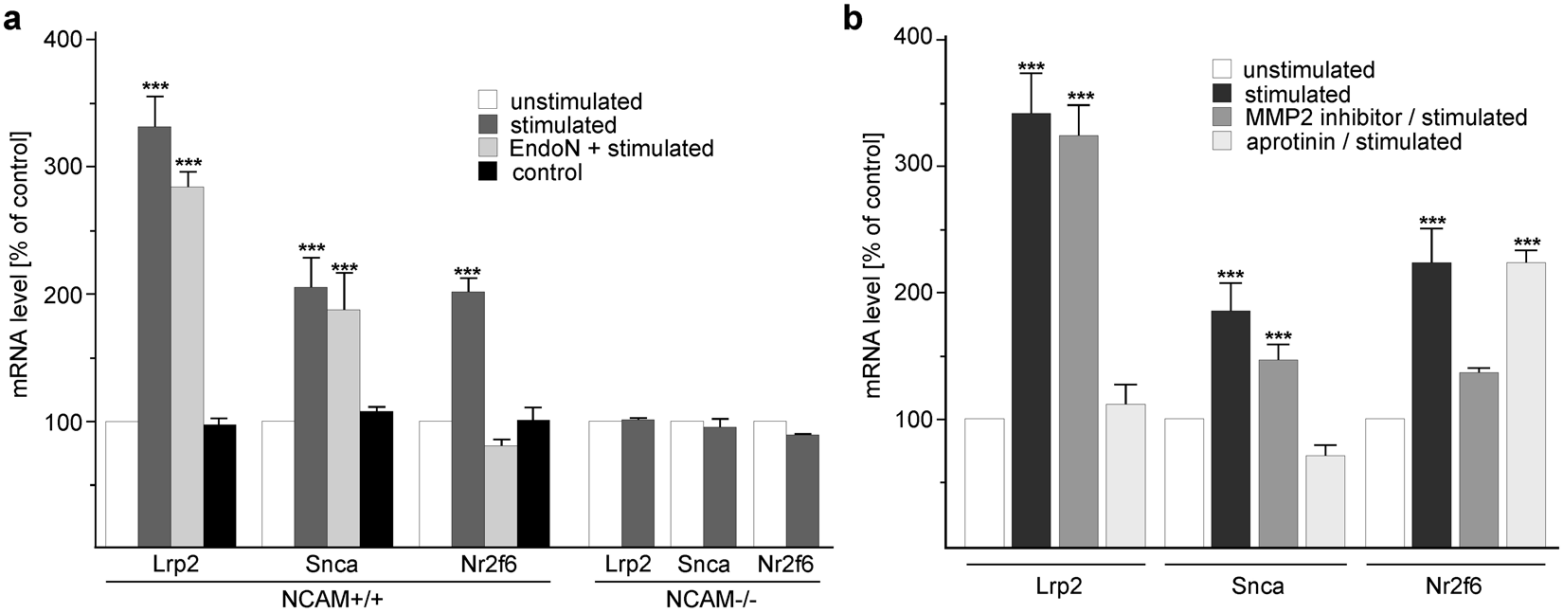
PSA-lacking and -carrying NCAM fragments regulate the expression of different genes. (**a**, **b**) Cerebellar neurons from wild-type (NCAM+/+) or NCAM-deficient (NCAM−/−) mice were not treated (unstimulated) or treated with function-triggering guinea pig NCAM antibody after pretreatment without (stimulated) or with EndoN (EndoN / stimulated), MMP2 inhibitor (MMP2 inhibitor / stimulated) or aprotinin (aprotinin / stimulated). For control, cerebellar neurons from wild-type mice were treated with function-triggering L1 antibody 557 (control). RNA was isolated and used for qPCR. Mean values and standard deviations from 3 independent experiments with triplicates per experiment (n = 9) are shown for the relative levels of Nr2f6, Snca and Lrp2 mRNAs relative to the levels in untreated cells (set to 100%). Differences between groups are indicated (***p < 0.001; two-way ANOVA with Bonferroni post-hoc test).

Since only the Nr2f6, Lrp2 and Snca genes were identified in two different sets of expression profiling experiments to be specifically regulated in their expression upon stimulation of cerebellar neurons with different NCAM antibodies, the NCAM-dependent regulation of expression of these genes was analysed in more detail. To test whether nuclear PSA-lacking NCAM fragment up-regulates expression of the Lrp2 and Snca mRNAs and PSA-carrying NCAM fragment up-regulates expression of the Nr2f6 mRNA, qPCR was carried out with Lrp2-, Snca- and Nr2f6-specific primers and with mRNA from dissociated cerebellar neurons treated with NCAM antibody after pretreatment without or with EndoN to remove PSA from NCAM. Relative to the mRNA levels in untreated neurons, elevated Lrp2, Snca and Nr2f6 mRNA levels were determined after stimulation of neurons with NCAM antibody (Fig. 4a). Enhanced Lrp2 and Snca mRNA levels were also found in NCAM antibody-treated neurons after removal of PSA by EndoN, whereas no enhanced Nr2f6 mRNA levels were observed in NCAM antibody-stimulated neurons after EndoN pretreatment (Fig. 4a). These results suggest that the regulation of Lrp2 and Snca gene expression depends on the nuclear PSA-lacking NCAM fragment, while the Nr2f6 gene expression is regulated by the nuclear PSA-carrying NCAM fragment.

The serine protease inhibitor aprotinin blocks the generation of the PSA-lacking NCAM fragment^25^, while an inhibitor of matrix metalloprotease 2 (MMP2) prevents the generation of the PSA-carrying NCAM fragment by different pathways^44^. Thus, these protease inhibitors were used to test whether the Lrp2 and Snca gene expression depend on the generation of the nuclear PSA-lacking NCAM fragment, while the Nr2f6 gene expression depends on the generation of the nuclear PSA-carrying NCAM fragment. To this aim, qPCR was performed using mRNA from cerebellar neurons pretreated in the presence and absence of aprotinin or a MMP2-specific inhibitor and treated without and with NCAM antibody. Relative to the levels in untreated neurons, enhanced Lrp2 and Snca mRNA levels were found in neurons stimulated with the NCAM antibody in the absence of both protease inhibitors and in the presence of the MMP2-specific inhibitor, but not in the presence of aprotinin (Fig. 4b), whereas enhanced Nr2f6 mRNA levels were observed in neurons after stimulation with the NCAM antibody in the absence of both protease inhibitors and in the presence of aprotinin, but not in the presence of the MMP2-specific inhibitor (Fig. 4b). These results confirm that expression of Lrp2 and Snca mRNA is up-regulated by the nuclear PSA-lacking NCAM fragment, while expression of the Nr2f6 mRNA is up-regulated by the PSA-carrying NCAM fragment.

**Figure 5:**
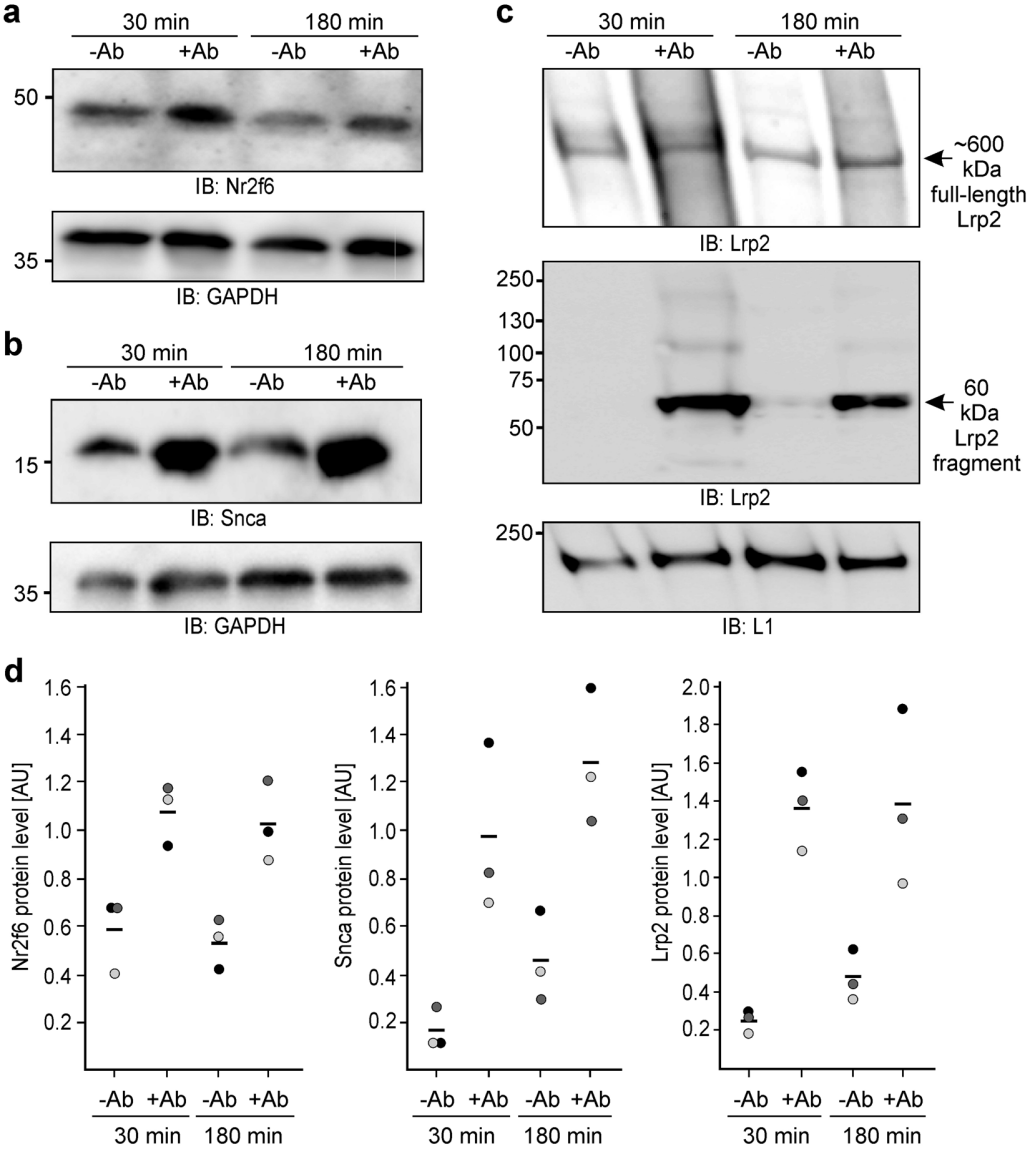
NCAM fragments without or with PSA regulate the protein expression of Snca and Lrp2 or Nr2f6. Cerebellar neurons from wild-type mice were treated for 30 or 180 min without (−Ab) or with function-triggering guinea pig NCAM antibody (+Ab). Cell lysates were subjected to immunoblot (IB) analysis with antibodies against Nr2f6 (**a**), Snca (**b**), Lrp2 (**c**) protein or against glyceraldehyde 3-phosphate dehydrogenase (GAPDH) (**a**, **b**) or L1 (**c**) to control gel loading. (**a**–**c**) Representative immunoblots out of three independent experiments are shown. For clarity, only parts of the blot with Nr2f6-, Lrp2-, Snca-, GAPDH-, and L1-positive bands are shown. The Lrp2 immunoblot (**c**) is shown after long exposure time (upper panel) and short exposure (lower panel). Full-length Lrp2 (~600 kDa) and the 60 kDa Lrp2 fragment are indicated by arrows. (**d)** Levels of Nr2f6 and Snca as well as the sums of the full-length Lrp2 and the 60 kDa Lrp2 fragment levels were quantified by densitometry and normalized to GAPDH or L1 levels. Single values (circles) and mean values (line) from 3 independent experiments are shown for the levels of Nr2f6, Snca and Lrp2 proteins.

Immunoblot analysis of lysates from cerebellar neurons after treatment with or without NCAM antibody for 30 or 180 min showed elevated expression of Nr2f6, Snca and Lrp2 protein in neurons after NCAM antibody treatment relative to non-treated neurons (Fig. 5a–d). Of note, the protein level of the full-length ~600 kDa Lrp2 was not significantly altered, whereas the level of the ~60 kDa proteolytic Lrp2 fragment^45^ was increased by treatment with the NCAM antibody (Fig. 5c). These results suggest that the expression of Snca protein and expression and subsequent proteolytic cleavage of full-length Lrp2 protein are up-regulated by the nuclear PSA-lacking NCAM fragment and that the expression of the Nr2f6 protein is up- regulated by the nuclear PSA-carrying NCAM fragment. Moreover, the enhanced levels of the Lrp2 fragment indicated that stimulation of neurons with NCAM antibody not only up-regulates the Lrp2 protein expression, but also concomitantly stimulates the proteolytic cleavage of full-length Lrp2.

### The diurnal expression of Nr2f6 mRNA correlates with the levels of the nuclear PSA-carrying NCAM fragment

**Figure 6:**
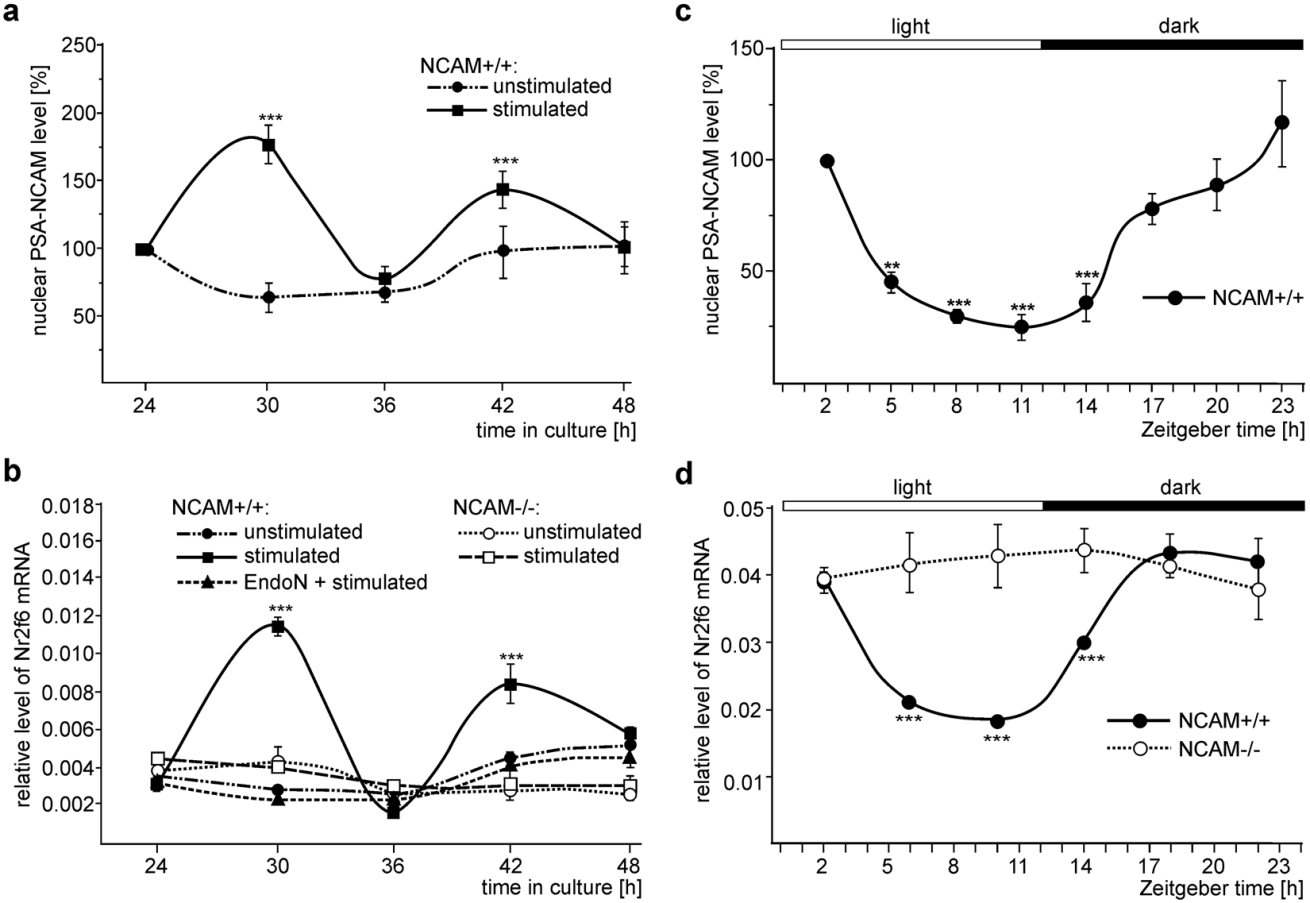
Nuclear PSA-NCAM affects the expression of Nr2f6 mRNA in dissociated cerebellar neurons and in cerebellar tissue. (**a**, **b**) Wild-type (NCAM+/+) (**a**, **b**) and NCAM-deficient (NCAM−/−) (**b**) cerebellar neurons were maintained in culture for different time periods and treated without (unstimulated) or with (stimulated) chicken NCAM antibody (**a**) after pretreatment without (stimulated) or with EndoN (EndoN + stimulated). (**a**) Nuclear fractions were subjected to immunoblot (IB) analysis with PSA and histone H3 antibodies. Mean values and standard deviation from 5 independent experiments are shown for the PSA levels normalized to histone H3 levels and relative to the normalized PSA level of non-treated cells after 24 h in culture (set to 100%) (***p < 0.001; two-way ANOVA with Dunnett’s multiple comparison test). (**b**) RNA was isolated and used for qPCR. Mean values and standard deviation from 3 independent experiments with triplicates (n = 9) are shown for the Nr2f6 mRNA level relative to the reference genes actin and tubulin (***p < 0.001; two-way ANOVA with Bonferroni post-hoc test). (**c**, **d)** Cerebella of 3-month-old wild-type (**c, d**) (NCAM+/+) or NCAM-deficient (NCAM−/−) (**d**) mice were isolated at different ZT points of the 12 h light/12 h dark cycle (ZT0: lights on) and subjected to isolation of nuclear fractions and immunoblot (IB) analysis with PSA and histone H3 antibodies (**c**) or to isolation of RNA and qPCR (**d**). (**c)** Mean values and standard deviations from 2 independent experiments with 3 males and 3 females per group (n = 12) are shown for the nuclear PSA levels normalized to histone H3 levels and relative to normalized PSA levels of the ZT2 group (set to 100%) (**p < 0.01, ***p < 0.001; two-way ANOVA with Dunnett’s multiple comparison test). (**d**) Mean values and standard deviations from 3 independent experiments with 2 males and 2 females per group (n = 12) are shown for the Nr2f6 mRNA levels relative to the level of the reference genes actin and tubulin. Differences relative to the levels at ZT2 are indicated (***p < 0.001; two-way ANOVA with Dunnett’s multiple comparison test).

Since Nr2f6 and PSA are involved in entrainment of circadian rhythm by regulating expression of clock-related genes^26,46^, we determined whether the alterations of nuclear PSA-NCAM levels during the circadian rhythm in cerebellar cell cultures and tissue are associated with changes in the Nr2f6 mRNA levels. Relative to the levels in non-treated neurons, nuclear PSA-NCAM levels were elevated by NCAM antibody treatment after 30 h or 42 h in culture, but not by antibody application after 24, 36 or 48 h (Fig. 6a). The enhanced nuclear PSA-NCAM levels coincided with increased levels of Nr2f6 mRNA (Fig. 6b). Recently, we showed that the enhanced nuclear PSA-NCAM levels coincided with a decreased mRNA level of the clock-related gene Circadian Locomotor Output Cycles Kaput (CLOCK) and scatter plot analysis revealed a negative correlation between the nuclear PSA-NCAM levels and the CLOCK mRNA levels (r^2^ = 0.7938)^26^. Scatter plot analysis of nuclear PSA-NCAM levels and Nr2f6 mRNA levels in neurons after antibody stimulation revealed a strong positive correlation between the nuclear PSA-NCAM levels and Nr2f6 mRNA levels (r^2^ = 0.9347). The mRNA levels of the clock-unrelated genes hypoxanthine phosphoribosyl transferase 1 and RNA polymerase subunit 2 in untreated and stimulated cerebellar neurons were similar and did not correlate with the nuclear PSA-NCAM levels (Supplementary Fig. S2). After removal of PSA, Nr2f6 mRNA levels were not enhanced by antibody stimulation (Fig. 6b), implying that PSA-NCAM, but not NCAM without PSA alters expression of this clock-related gene. Of note, antibody application did not alter Nr2f6 mRNA levels in NCAM-deficient neurons (Fig. 6b).

In cerebellar tissue, we recently showed a moderate negative correlation between the nuclear PSA-NCAM levels and the mRNA levels of the clock-related gene Period-1 (Per-1) during the circadian rhythm (r^2^ = 0.6057): low and high nuclear PSA-NCAM levels at the end of the light phase and of the dark phase are associated with increased and decrease Per-1 mRNA levels, respectively^26^. The decrease of nuclear PSA levels during the light phase correlated with a decline in the Nr2f6 mRNA levels, and the increase of nuclear PSA levels during the dark phase was associated with a rise in the Nr2f6 mRNA levels (Fig. 6c, d). The mRNA levels of the clock-unrelated gene glyceraldehyde 3-phosphate dehydrogenase did not change during the circadian rhythm (Supplementary Fig. S3). Scatter plot analysis of nuclear PSA-NCAM levels and Nr2f6 mRNA levels showed a positive correlation between the nuclear PSA-NCAM levels and Nr2f6 mRNA levels during the circadian rhythm (r^2^ = 0.7144). In comparison to the cerebellum of wild-type mice, the cerebellum of NCAM-deficient mice displayed no significant changes in expression of Nr2f6 mRNA levels (Fig. 6d).

Since Nr2f6 mRNA levels were not enhanced by NCAM antibody treatment in cultured wild-type neurons after removal of PSA or in NCAM-deficient neurons and since no alterations in Nr2f6 mRNA levels during the circadian rhythm were observed in NCAM-deficient cerebella, we conclude that the expression of Nr2f6 during the circadian rhythm depends on PSA-carrying NCAM.

## Discussion

In a previous study we showed that the nuclear import of the PSA-lacking NCAM fragment is mediated by calmodulin^25^. Here, we show that the nuclear import of the PSA-carrying NCAM fragment involves cofilin and PC4. Co-immunoprecipitation of PSA with cofilin and PC4 from nuclear fractions and co-localization of PSA with cofilin and PC4 in nuclei of cerebellar neurons in culture and in tissue suggests that PSA-NCAM interacts with cofilin and PC4 in the cell nucleus. Since nuclear cofilin plays a role in the elongation phase of RNA polymerase II-dependent transcription^30^ and since PC4 activates RNA polymerase II-dependent transcription^37^, it is conceivable that the interactions of PSA with PC4 and cofilin in the nucleus stimulate RNA polymerase II-mediated transcription. Since stimulation of cerebellar neurons with function-triggering NCAM antibody enhances the levels of acetylated histone H3 at position Lys9, being associated with enhanced transcription levels, we proposed that the generation and nuclear import of PSA-carrying and PSA-lacking NCAM fragments regulate transcription in distinct manners. Here, we provide evidence that the nuclear PSA-carrying NCAM fragment up-regulates the expression of Nr2f6 mRNA and protein, while the nuclear PSA-lacking NCAM fragment up-regulates the mRNA and protein expression of Lrp2 and Snca. An approximate two-fold increase in Nr2f6 mRNA and protein levels was observed, whereas the approximate two- or three-fold increase in Snca or Lrp2 mRNA levels coincide with an approximate five- or six-fold increase in Snca or Lrp2 protein levels, respectively. These differences in mRNA versus protein levels may indicate that stimulation of NCAM-dependent signal transduction pathways or nuclear NCAM increases translation and/or stability of Snca or Lrp2 proteins. Nr2f6 protein is an orphan nuclear receptor and a transcription factor which represses transcription and modulates hormonal responses and which is required for development of the locus coeruleus and entrainment of the forebrain circadian clock^46^. We now show that nuclear PSA-NCAM is involved in the regulation of the Nr2f6 gene expression during the circadian rhythm, supporting the notion that the nuclear PSA-carrying NCAM fragment affects regulation of the circadian rhythm^26^. The nuclear PSA-lacking NCAM fragment enhances the expression of Lrp2 mRNA and of Lrp2 protein which is a member of the low-density-lipoprotein (LDL) receptor family of lipoprotein receptors and which is essential for development and functional homeostasis of the adult nervous system. Lrp2 protein acts as a multi-ligand endocytic receptor that binds to a variety of different ligands, such as proteases, protease inhibitors, vitamin-binding proteins, hormones, hormone-binding proteins, morphogens, signal transducing molecules, extracellular matrix proteins, lipoproteins, and general nutrients^47–49^. During nervous system development, Lrp2 plays crucial roles in proliferation and differentiation of neural precursor cells, neuronal survival, and neurite outgrowth and branching^47–49^. In the adult nervous system, Lrp2 is involved in neurogenesis, regulation of the cholesterol metabolism and clearance of amyloid-β which is associated with Alzheimer´s disease^47–49^. In addition, Lrp2 has been genetically linked to Alzheimer’s disease^47–49^. Interestingly, NCAM mediates induction of signal transduction pathways, affects neurite outgrowth and branching as well as synaptogenesis during development. It is also involved in synaptic plasticity and associated with neuropsychiatric and neurodegenerative disorders^3^. It is interesting in this context that overexpression of NCAM in the adult mouse brain induces neurogenesis^50^ and reduced NCAM levels have been reported in brains of Alzheimer´s disease patients^51^. Based on these findings, it is tempting to speculate that regulation of Lrp2 expression by nuclear PSA-lacking NCAM contributes to regulating Lrp2-mediated neural functions and Alzheimer´s disease pathology. Of note, the nuclear PSA-lacking NCAM fragment also enhances the expression of Snca mRNA and protein. Snca protein is presynaptically active, responsible for synaptic vesicle trafficking and release, synaptic transmission, and for biosynthesis, release, transport and reuptake of dopamine, and it is linked to Parkinson’s disease^52,53^. Since NCAM contributes to synaptic vesicle recycling^54^ and synaptic transmission^3^, it is conceivable that regulation of Snca expression by nuclear PSA-lacking NCAM also contributes to regulating Snca-mediated functions which are abnormal in Parkinson´s disease.

The results of the present study indicate that the nuclear PSA-carrying NCAM fragment contributes to the regulation of gene expression during the circadian rhythm, while the nuclear PSA-lacking NCAM fragment is not dependent in its effect on the circadian rhythm and affects several essential functions in the developing and adult brain, such as lipid metabolism, hormone homeostasis, dopamine turnover or synaptic plasticity. Thus, the differential regulation of gene expression by nuclear NCAM fragments with and without PSA emphasizes the independence of the PSA-associated functions from functions of its carrier NCAM.

## Methods

### Animals

NCAM-deficient mice^55^ and their wild-type littermates as well as C57BL/6 J mice of both sexes were used in all experiments which were conducted in accordance with the German and European Community laws on protection of experimental animals and approved by the responsible committee of the State of Hamburg (animal permit number ORG 679 Morph). Mice were maintained as described^22^.

At different time points of the 12 h light/12 h dark cycle (Zeitgeber time (ZT) 2, 6, 10, 14, 18 and 22 or 2, 5, 8, 11, 14, 17, 20 and 23 relative to ZT0 which is defined as lights on at 7.00 pm), cerebella were removed from the 3-month-old adult males and females and subjected either to subcellular fractionation using the Subcellular Protein Fractionation Kit for Tissues (ThermoFisherScientific, Darmstadt, Germany) or to isolation of RNA using Qiashredder and RNAeasy Plus Kit (Qiagen, Hilden, Germany). Similar results were obtained for males and females.

### Antibodies and reagents

Polyclonal rabbit antibodies against the extracellular domains of NCAM, L1 or CHL1 and monoclonal L1 antibody 557 have been described^56–58^. The polyclonal chicken or guinea pig antibodies against the extracellular domain of mouse NCAM were produced by Pineda (Berlin, Germany) using a recombinant fusion protein of the extracellular NCAM domain and the Fc part of human IgG^21^ as antigen. The mouse monoclonal PSA antibody 735^59^ and Endo N^60^ were kind gifts of Rita Gerardy-Schahn (Zentrum Biochemie, Institut für Zelluläre Chemie, Medizinische Hochschule, Hannover, Germany). Rabbit PC4 antibody (ab84459) used for immunoblot analysis, function blocking and proximity ligation assay as well as α-synuclein antibody (ab131508) were from Abcam (Cambridge, UK) and rabbit cofilin antibody (#3312) used for immunoblot analysis and proximity ligation assay was from Cell Signaling Technology (New England Biolabs, Frankfurt). Antibodies against histone H3 (C-16; sc-8654), glyceraldehyde 3-phosphate dehydrogenase (GAPDH) (FL-335; sc25778), Nr2f6 (EAR2; L-12; sc-23229), Lrp2 (megalin; G-9; sc-515750) and cofilin (FL-166; sc-33779) used for function blocking were from Santa Cruz Biotechnology (Heidelberg, Germany). Secondary antibodies were from Dianova (Hamburg, Germany). MMP-2 inhibitor III (CAS 704888-90-4) and aprotinin were from Merck Chemicals (Darmstadt, Germany). The polyclonal antibodies against dimethyl histone H3-K9 (A-4035–025), trimethyl histone H3-K9 (A-4033-025) and acetyl histone H3-K9/14 (A-4021-025) and the MethylFlash Methylated DNA Quantification and MethylFlash Hydroxymethylated DNA Quantification kits were from Epigentek (Farmingdale, NY, USA). Purified recombinant human cofilin^61,62^ was a kind gift of James Bamburg (Department of Biochemistry and Molecular Biology, Colorado State University, Fort Collins, CO, USA). GST-tagged murine cofilin and untagged cofilin^63^ were kind gifts of Jan Faix (Department of Biophysical Chemistry, Medizinische Hochschule, Hannover, Germany). His-tagged full-length PC4 and His-tagged PC4 mutants with deletion of amino acids 1–87 or 62–127^31^ were kind gifts of Tapas Kundu (Transcription and Disease Laboratory, Molecular Biology and Genetics Unit, Jawaharlal Nehru Centre for Advanced Scientific Research, Bangalore, India).

Histone H2A (#14-493) and H2B (#14-491) were from Merck Chemicals and glyceraldehyde-3-phosphate dehydrogenase and colominic acid (C5762) were from Sigma-Aldrich (Steinheim, Germany).

### Immunoblot analysis, proximity ligation assay, co-immunoprecipitation, silver staining and mass spectrometry

SDS-PAGE with 4–20% Criterion™ Tris-HCl Gels or 4–20% Mini-Protean TG™ Precast Protein Gels (Bio-Rad, Munich, Germany) and immunoblot analysis using chemiluminescent substrates as well as proximity ligation assay have been described^26^. NuPAGE Tris-Acetate Precast Gels and NuPAGE Transfer buffer (ThermoFisher Scientific) were used for immunoblot analysis of Lrp2. Chemiluminescence was monitored using ImageQuantTM LAS 4000mini (GE Healthcare, Freiburg, Germany) and band intensities were quantified using ImageJ software. Intensities relative to loading controls were calculated and examined by one-way ANOVA followed by Dunnett´s multiple comparison test. Silver staining, mass spectrometry and co-immunoprecipitation have been described^64^.

### Affinity chromatography

For isolating a fraction enriched in alkaline-solubilized nuclear proteins, brains were homogenized as described^38^. The homogenate was centrifuged at 1,000 × g at 4 °C and the resulting pellet was incubated for 30 min on ice in alkaline extraction buffer (25 mM Tris-HCl, 150 mM NaCl, 5 mM EDTA, 150 mM NaHCO_3_, pH 11.5) followed by centrifugation at 100,000 × g for 30 min at 4 °C. After adjusting the pH to 7.4 using HCl, the supernatant was applied to a column containing immobilized PSA-mimicking anti-idiotypic single chain variable fragment antibody selected by phage display for binding to the PSA binding site on PSA-specific antibody 735 and thus representing an anti-idiotype receptor for the PSA ligand^38,39^. Bound proteins were eluted as described^38,39^.

### ELISA

ELISA was performed as described^38^. Briefly 25 µl of cofilin, PC4 proteins, glyceraldehyde-3-phosphate dehydrogenase (20 µg/ml in PBS, pH 7.4), histone H2A, histone H2B (10 µg/ml in PBS, pH 7.4) or PBS alone were incubated at 4 °C overnight in 384-well Corning^®^ non-treated microtiter plates (Corning, Lowell, MA, USA). After blocking with 2% BSA in PBS for 1.5 h, the wells were washed once with PBS containing 0.05% Tween-20 (PBST) and incubated with increasing concentrations of colominic acid for 1 h at room temperature. After washing three times with PBST and once with PBS, PSA antibody (25 µl, 5 µg/ml) was added and incubated for 1 h at room temperature. Plates were then washed three times with PBST, incubated with horseradish peroxidase (HRP)-conjugated secondary mouse antibody (25 µl, 1:2,000) for 1 h at room temperature and washed three times with PBST. Binding was visualized using o-phenylenediamine dihydrochloride (ThermoFisher Scientific), the reaction as stopped using 1.4 M H_2_SO_4_ and absorption was read using an ELISA reader at 492 nm (Bio-TEK, Winooski, VT, USA).

### Culture of cerebellar granule neurons

Cerebellar granule neurons were prepared and maintained as described^26^. Briefly, at 8.00 am (lights on at 7.00 am) cerebellar granule neurons were prepared from 6- to 8-day-old C57Bl/6 J mice and maintained in 6-well plates in serum-free medium for 30 h or for the indicated time periods. Cells were then pretreated without or with Endo N (25 units per well), aprotinin (1 µM), MMP-2 inhibitor III (25 nM) for 1 h and treated without or with chicken or guinea pig NCAM antibody (100 µg protein/ml) or monoclonal rat antibody 557 against L1 (as control) for 30 min in the culture incubator. The treated neurons were subjected to subcellular fractionation using the Subcellular Protein Fractionation Kit for Cultured Cells (ThermoFisher Scientific) or were processed for isolation of RNA using Qiashredder and RNAeasy Plus Kit (Qiagen).

### Microarray analysis

In three independent experiments, dissociated cerebellar neurons were maintained for 30 h in serum-free culture medium, pretreated without or with EndoN (25 units per well) for 1 h and thereafter treated without or with chicken NCAM antibody (400 µg of antibody precipitated from egg yolk by polyethylene glycol) for 15 min in the culture incubator. In three different independent experiments, dissociated cerebellar neurons were maintained for 30 h in serum-free culture medium, incubated without or with aprotinin (1 µM) for 30 min and then treated without or with 25 µg IgG/ml of polyclonal rabbit NCAM antibody, polyclonal rabbit CHL1, polyclonal rabbit L1 antibody, rat monoclonal L1 antibody 557, non-immune rabbit antibody or non-immune mouse antibody for 5 h in the culture incubator. Cells from two culture wells per treatment and experiment were pooled for isolation of total RNA using QIAshredder and RNeasy kit according to the manufacturer’s instructions (Qiagen). Total RNA concentrations and absorption ratios 260/280 nm were determined using the Nanodrop ND-1000 spectrophotometer (ThermoFisher Scientific). RNA sample quality control, cDNA synthesis and labelling, hybridization using Affymetrix GeneChip Mouse Genome 430 2.0 arrays and signal detection were performed by the Analytical and Informatics Services at the Rutgers University Cell and DNA Repository (RUCDR) in accordance with the manufacturer’s protocol (Affymetrix, Santa Clara, CA, USA).

For data and statistical analysis and data visualization, extraction of expression values from the CEL file, GC-RMA (robust multi-array average) and quantile normalization and pairwise differential expression analysis using a standard t-test with a Benjamini and Hochberg multiple testing correction were performed using the Geospiza Genesifter software package (PerkinElmer, Waltham, MA, USA). The exploratory list was generated by selecting genes with a t-test P-value of < 0.05. Normalized log values of signal intensities from each group were compared to those of the other groups, and genes showing differences in signal intensities between groups were listed by average fold-change expression with SEM (calculated by taking the antilog of log values).

***qPCR***. For reverse transcription, oligoT_18_ primer and M-MLV reverse transcriptase (Sigma-Aldrich) were used. qPCR was performed in triplicates using reverse transcribed mRNA, the 7900HT Fast Real-Time PCR System (ThermoFisher Scientific), the qPCR kit SYBR^®^ Green I, ROX (Eurogentec, Cologne, Germany) and primers for determination of the mRNA levels of Nr2f6 (5′-GAA GCA CTT CTT GAG CCG AC-3′, 5′-AAT CTC AGC TAC ACC TGC CG-3′), Lrp2 (5′-GGC AGT GGG AAT TTT CGC TG-3′, 5′-CAG GAG CTA GGG ATG CAG G -3′) and Snca (5′-CAG GCA TGT CTT CCA GGA TT -3′, 5′- GGG AAT ATA GCT GCT GCC AC -3′), or of the reference genes actin (5′- TCC TGT GGC ATC CAT GAA ACT -3′, 5′-TTC TGC ATC CTG TCA GCA ATG-3′), tubulin (5′-CGC ACG ACA TCT AGG ACT GA-3′, 5′-TGA GGC CTC CTC TCA CAA GT-3′), or of the clock-unrelated control gene hypoxanthine phosphor ribosyltransferase 1 (5′-GTT CTT TGC TGA CCT GCT GGA-3′, 5′-TCC CCC GTT GAC TGA TCA TT-3′), RNA polymerase subunit 2 (5′-CCA ATG ATA TTG TGG AGA TCT TCA C-3′, 5′-GGC ATG TCA TAG TGT CAC ACA GGA-3′) and glyceraldehyde 3-phosphate dehydrogenase (5′-AGC CTC GTC CCG TAG ACA AAA-3′, 5′- GGC AAT CTC CAC TTT G-3′). The SDS 2.4 software was taken for analysis of the qPCR data. The mRNA levels of Nr2f6, Lrp2 and Snca relative to the mRNA levels of the reference genes were calculated. Data of relative gene expression at mRNA level in cultured cerebellar neurons were analysed by two-way analysis of variance (ANOVA) followed by Bonferroni post-hoc test.

### Scatter plot analysis

Mean values for relative gene expression and for nuclear PSA levels were calculated and arranged in a scatter plot as described^26^. Linear regression was performed for correlation analysis and r^2^ values of > 0.3, 0.3–0.5 and < 0.5 were considered to indicate low, moderate or high correlation.

### Data Availability

All data generated or analysed during this study are included in this published article (and its Supplementary Information files).

## Electronic supplementary material

Supplementary Figures and Tables
