# Supplementary material for: Nuclear fragments of the neural cell adhesion molecule NCAM with or without polysialic acid differentially regulate gene expression
Source: Sci Rep. 2017 Oct 19;7:13631. doi: 10.1038/s41598-017-14056-x (PMC5648764; doi:10.1038/s41598-017-14056-x)
Supplement: Supplementary file 1 — Supplementary Figures and Tables [file 41598_2017_14056_MOESM1_ESM.pdf]

Supplementary Information

Nuclear fragments of the neural cell adhesion molecule NCAM with or without polysialic acid regulate expression of different genes

by

Nina Westphal, Thomas Theis, Gabriele Loers, Melitta Schachner and Ralf Kleene

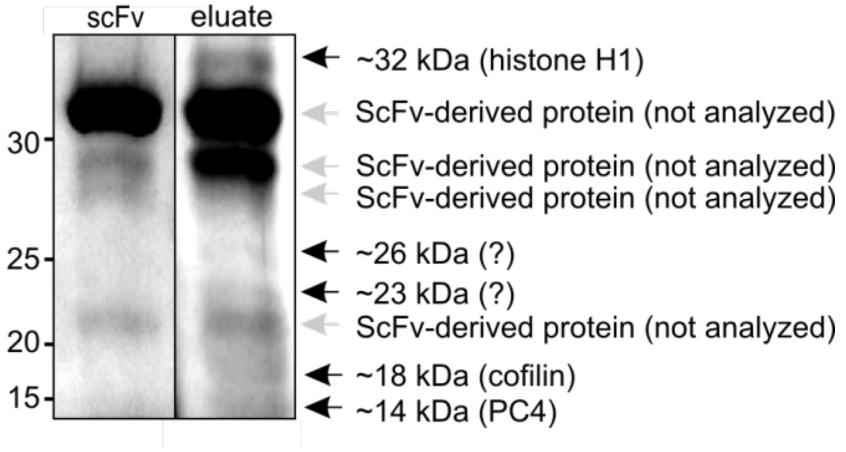

Figure S1. **Identification of cofilin and PC4 as novel PSA-binding proteins**. An alkaline-soluble nuclear protein fraction from brains was used for affinity chromatography with immobilized PSA-mimicking scFv antibody. Proteins binding to the scFv antibody (eluate) were subjected to silver staining together with the scFv antibody (scFv) preparation used for affinity chromatography. The image of the silver-stained gel is shown before cutting out protein bands for mass spectrometric analysis. The lanes were not adjacent to each other but derived from the same gel. Only the lower part of the gel with silver-stained protein bands is shown. The grey arrows indicate proteins in the eluate which were also seen in the scFv antibody preparation. These proteins were considered as scFv antibody-derived proteins and were not subjected to mass spectrometry. The black arrows indicate diffusely silver-stained ~14, ~18, ~23, ~26 and ~32 kDa proteins which were seen in the eluate and which were not or only hardly detectable in the scFv antibody preparation. These proteins were subjected to mass spectrometry. The ~14, ~18 and ~32 kDa proteins were identified as PC4, cofilin-1 and histone H1, respectively. The ~23 and ~26 proteins could not be assigned to a certain protein.

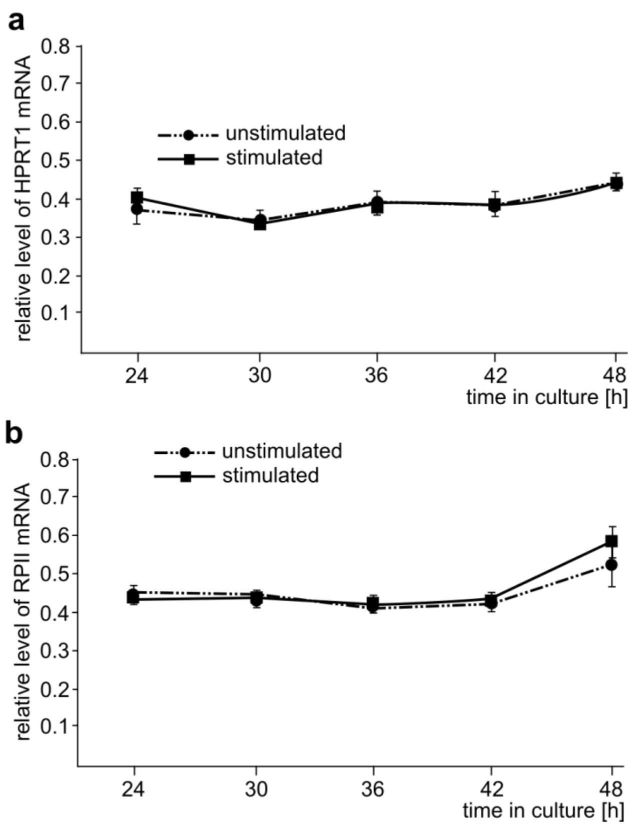

Figure S2. **Nuclear PSA-NCAM does not affect the mRNA expression of clock-unrelated genes in dissociated cerebellar neurons**. Wild-type cerebellar neurons were maintained in culture for different time periods and treated without (unstimulated) or with guinea pig NCAM antibody (stimulated). RNA was isolated and used for qPCR. Mean values and standard deviation from 3 independent experiments with triplicates (n=9) are shown for the hypoxanthine phosphoribosyl transferase 1 (HPRT1) (**a**) and RNA polymerase subunit 2 (RPII) (**b**) mRNA levels relative to the reference genes actin and tubulin.

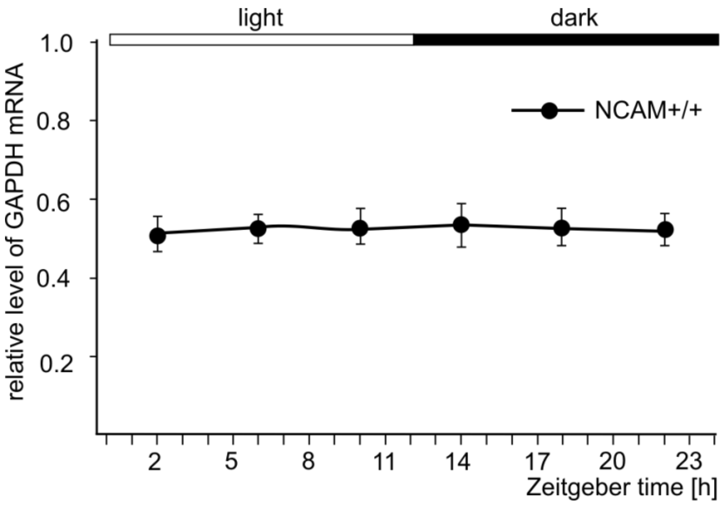

Figure S3. **Nuclear PSA-NCAM does not affect the mRNA expression of the clock-unrelated gene glyceraldehyde 3-phosphate dehydrogenase in cerebellar tissue during the circadian rhythm**. Cerebella of 3-month-old wild-type mice were isolated at different ZT points of the 12 h light/12 h dark cycle (ZT0: lights on) and subjected to isolation of RNA and qPCR. Mean values and standard deviations from 2 independent experiments with 2 males and 2 females per group (n=8) are shown for the glyceraldehyde 3-phosphate dehydrogenase mRNA levels relative to the level of the reference genes actin and tubulin.

Gene ID	stim vs unstim	stim vs EndoN/stim	Gene name
Abi1	1.155±0.013	1.215±0.012	abelson interactor 1
Actl6a	1.286±0.009	1.235±0.009	actin-like 6A
Arfgap1	1.270±0.012	1.229±0.009	ADP-ribosylation factor GTPase activating protein 1
Asb6	1.216±0.005	1.228±0.004	ankyrin repeat and SOCS box-containing 6
Asxl1	1.236±0.021	1.247±0.019	additional sex combs like 1
Atg4c	1.268±0.020	1.249±0.020	autophagy-related 4C
Cnnm2	1.234±0.018	1.262±0.017	cyclin M2
CNOT10	1.349±0.008	1.259±0.009	CCR4-NOT transcription complex, subunit 10
CPPED1	1.373±0.009	1.212±0.009	calcineurin-like phosphoesterase domain containing 1
Heatr5b	1.267±0.020	1.238±0.022	HEAT repeat containing 5B
Ints2	1.282±0.013	1.262±0.012	integrator complex subunit 2
Lrrtm3	1.262±0.012	1.375±0.010	leucine rich repeat transmembrane neuronal 3
Mtfr1	1.221±0.016	1.252±0.017	mitochondrial fission regulator 1
Naalad2	1.388±0.016	1.309±0.018	N-acetylated alpha-linked acidic dipeptidase 2
Napa	1.254±0.007	1.266±0.007	N-ethylmaleimide sensitive fusion protein attachment protein, α
Npat	1.276±0.018	1.227±0.021	nuclear protein in the AT region
Nr2f6	1.320±0.023	1.349±0.025	nuclear receptor subfamily 2, group F, member 6
Pcdh10	1.422±0.012	1.408±0.012	OL-protocadherin isoform
Pggt1b	1.210±0.021	1.226±0.020	protein geranylgeranyltransferase type I, beta subunit
Pias2	1.296±0.027	1.274±0.028	protein inhibitor of activated STAT 2
Pigt	1.256±0.020	1.218±0.023	phosphatidylinositol glycan anchor biosynthesis, class T
Pitrm1	1.221±0.022	1.267±0.019	pitrilysin metallepetidase 1
Scn3b	1.281±0.017	1.244±0.020	sodium channel, voltage-gated, type III, β, transcript variant 1
Sfi1	1.314±0.011	1.240±0.011	Sfi1 homolog, spindle assembly associated
Slc25a16	1.236±0.021	1.244±0.020	solute carrier family 25, member 16
SLC25A39	1.314±0.017	1.270±0.018	solute carrier family 25, member 39
Tm2d2	1.205±0.006	1.209±0.020	TM2 domain containing 2
TSPYl3	1.247±0.070	1.273±0.006	TSPY-like 3
Tsr2	1.234±0.012	1.255±0.012	pre-rRNA-processing protein TSR2 homolog
Ttc7b	1.240±0.018	1.256±0.021	tetratricopeptide repeat domain 7B, transcript variant 1
UCK2	1.316±0.015	1.410±0.015	uridine-cytidine kinase 2
Zbtb45	1.303±0.050	1.306±0.006	zinc finger and BTB domain containing 45
Zdhhc20	1.279±0.011	1.278±0.012	zinc finger, DHHC domain containing 20
Zfp800	1.381±0.022	1.452±0.018	zinc finger protein 800
Zfyve1	1.366±0.002	1.200±0.002	zinc Finger, FYVE Domain containing 1
Zfyve26	1.247±0.024	1.287±0.024	zinc finger, FYVE domain containing 26
Zzz3	1.241±0.010	1.219±0.010	zinc finger, ZZ domain containing 3

Supplementary Table S1. NCAM-mediated PSA-dependent up-regulation of gene expression. The table shows the fold increase in levels of certain mRNAs in neurons treated with chicken NCAM antibody (stim) relative to levels in untreated cerebellar neurons (unstim) or in neurons treated with chicken NCAM antibody after pretreatment with EndoN (EndoN/stim) to remove PSA from NCAM. mRNAs are indicated by gene ID and name.

Gene ID	stim vs unstim	stim vs EndoN/stim	Gene name
Abcc5	1.308±0.010	1.202±0.010	ATP-binding cassette protein
Bcr	1.324±0.026	1.220±0.023	breakpoint cluster region
Cds2	1.371±0.046	1.315±0.040	CDP-diacylglycerol synthase 2
Chst3	1.276±0.012	1.241±0.011	chondroitin 6/keratan sulfotransferase 3
Ddef2	1.225±0.005	1.264±0.005	development and differentiation enhancing factor 2
Diap1	1.226±0.027	1.201±0.026	diaphanous homolog 1
Dnase1	1.217±0.024	1.248±0.023	deoxyribonuclease I
EBPL	1.250±0.017	1.237±0.020	emopamil binding protein-like
Eif4ebp2	1.319±0.039	1.301±0.027	eukaryotic translation initiation factor 4E binding protein 2
Elavl3	1.291±0.015	1.230±0.013	RNA-binding protein mHuC-S
Gats	1.210±0.011	1.247±0.011	opposite strand transcription unit to Stag3
Gnas	1.386±0.018	1.306±0.018	guanine nucleotide binding protein, α
Gpr39	1.310±0.024	1.249±0.026	G protein-coupled receptor 39
Hnrnpl	1.449±0.023	1.370±0.025	heterogeneous nuclear ribonucleoprotein L
Iqgap1	1.204±0.022	1.227±0.021	IQ motif containing GTPase activating protein 1
Kctd10	1.377±0.026	1.226±0.020	potassium channel tetramerisation domain containing 10
Kirrel3	1.255±0.014	1.482±0.013	Kin of IRRE like 3
L1cam	1.254±0.019	1.270±0.018	L1 cell adhesion molecule
Mapt	1.500±0.053	1.314±0.040	microtubule binding protein tau
NUDT7	1.270±0.022	1.269±0.018	nucleoside diphosphate linked moiety X type motif 7
Pik3r1	1.311±0.015	1.202±0.012	phosphatidylinositol 3-kinase, regulatory subunit 1
Ppara	1.233±0.029	1.215±0.032	peroxisome proliferator activated receptor alpha
Ppt1	1.311±0.026	1.252±0.022	palmitoyl-protein thioesterase
Prpf6	1.265±0.034	1.350±0.037	PRP6 pre-mRNA splicing factor 6 homolog
Rbm9	1.418±0.036	1.332±0.037	RNA binding motif protein 9
RHAMM	1.233±0.029	1.284±0.028	hyaluronan mediated motility receptor (RHAMM)
Rsrc2	1.250±0.023	1.367±0.021	arginine/serine-rich coiled-coil 2
Slc39a10	1.270±0.008	1.212±0.007	solute carrier family 39, member 10
Slc6a6	1.315±0.030	1.257±0.042	sodium-dependent taurine transporter
Smad5	1.226±0.029	1.210±0.028	MAD homolog 5
Usp7	1.259±0.011	1.229±0.024	ubiquitin specific peptidase 7
Xpo7	1.221±0.026	1.258±0.026	exportin 7
Zbtb46	1.289±0.023	1.215±0.023	zinc finger and BTB domain containing 46
Zfp597	1.330±0.005	1.204±0.006	zinc finger protein 597

Supplementary Table S2. NCAM-mediated PSA-dependent down-regulation of gene expression. The table shows the fold decrease in levels of certain mRNAs in neurons treated with chicken NCAM antibody (stim) relative to levels in untreated cerebellar neurons (unstim) or in neurons treated with chicken NCAM antibody after pretreatment with EndoN (EndoN/stim) to remove PSA from NCAM. mRNAs are indicated by gene ID and name.

Gene ID	stim vs unstim	EndoN/stim vs unstim	Gene name
Ccdc88c	1.154±0.005	1.309±0.027	coiled-coil domain containing 88C
Csmd1	1.174±0.002	1.289±0.015	CUB and Sushi multiple domains 1
Dlgap1	1.245±0.011	1.199±0.007	discs, large homolog-associated protein 1
Lrp2	1.415±0.021	1.267±0.011	low density lipoprotein receptor protein 2
Snca	1.251±0.010	1.249±0.021	synuclein, alpha
Trappc9	1.282±0.013	1.225±0.007	trafficking protein particle complex 9
Zfp40	1.329±0.014	1.157±0.004	zinc finger protein 40

Supplementary Table S3. NCAM-mediated PSA-independent up-regulation of gene expression. The table shows the fold increase in levels of certain mRNAs in neurons treated with chicken NCAM antibody after pretreatment without EndoN (stim) or with EndoN (EndoN/stim) relative to levels in untreated cerebellar neurons (unstim). mRNAs are indicated by gene ID and name.

Gene ID	stim vs unstim	EndoN/stim vs unstim	Gene name
Arsk	1.282±0.023	1.372±0.036	arylsulfatase K
B4galt5	1.384±0.013	1.319±0.020	UDP-Gal:βGlcNAc β1,4-galactosyltransferase, 5
Bach1	1.333±0.020	1.352±0.033	BTB and CNC homology 1
Bcl11b	1.365±0.038	1.350±0.052	B-cell leukemia/lymphoma 11B
Crip1	1.476±0.064	1.470±0.056	cysteine-rich protein 1
Dleu7	1.380±0.039	1.542±0.015	deleted in lymphocytic leukemia 7
Fam55c	1.482±0.026	1.373±0.012	family with sequence similarity 55, member C
Golt1b	1.325±0.013	1.304±0.021	Golgi transport 1 homolog B
Ifi30	1.473±0.066	1.306±0.002	interferon gamma inducible protein 30
Narf	1.401±0.014	1.421±0.021	nuclear prelamin A recognition factor
Nup133	1.285±0.037	1.311±0.032	nucleoporin 133
Phf17	1.422±0.024	1.244±0.029	PHD finger protein 17
Rnf114	1.398±0.014	1.280±0.023	zinc finger protein 313
Sel1l	1.392±0.035	1.324±0.027	Sel-1 suppressor of lin-12-like
Sucla2	1.336±0.032	1.263±0.015	succinate coenzyme A ligase, β subunit

Supplementary Table S4. NCAM-mediated PSA-independent down-regulation of gene expression. The table shows the fold decrease in levels of certain mRNAs in neurons treated with chicken NCAM antibody after pretreatment without EndoN (stim) or with EndoN (EndoN/stim) relative to levels in untreated cerebellar neurons (unstim). mRNAs are indicated by gene ID and name.
